# Eruption pattern of the maxillary canines: features of natural eruption seen in PTG at the late mixed stage—Part I

**DOI:** 10.1007/s40368-021-00650-1

**Published:** 2021-07-14

**Authors:** J. Ristaniemi, W. Rajala, T. Karjalainen, E. Melaluoto, J. Iivari, P. Pesonen, R. Lähdesmäki

**Affiliations:** 1 Research Unit of Oral Health Sciences, Oral Development and Orthodontics, Faculty of Medicine, University of Oulu, Oulu, Finland; 2 Infrastructure for Population Studies, Faculty of Medicine, University of Oulu, Oulu, Finland; 3 Oral and Maxillofacial Department, Medical Research Center Oulu (MRC Oulu), Oulu University Hospital, Oulu, Finland

**Keywords:** Dental age, Developing dentition, Human, Panoramic radiograph, Permanent tooth, Root formation

## Abstract

**Aim:**

To describe the variation of eruption pattern of maxillary canines in the late mixed stage of dentition seen in PTG when eruption was later natural.

**Methods:**

Material for this longitudinal and retrospective register-based study consisted of 1454 PTGs of children living in Eastern Finland (mean age 9.3 years, SD 0.6). Natural eruption of a canine consisted of 744 PTGs (336 girls and 408 boys) including 1488 maxillary canines. The variables examined were treatment/natural eruption, overlapping, inclination, dental age, developmental stage of the canine and lateral incisor.

**Results:**

Only 2.0% of maxillary canines had clear overlapping and 56.2% no overlapping was detected at the age of 8.5–10.5 years. Large inclination angle (≥ 25°) was found for 5.5% of examined canines. Overlapping of canine with lateral incisor root decreased as the development of canine root exceeded 1/3. Larger inclinations occurred at earlier stages but decreased significantly as the root developed from 1/3 to 1/2. Mean inclination was significantly larger at children with normal dental age and/or incomplete lateral incisors when overlapping occurred. Regardless overlapping mean inclination was larger if dental age was delayed and/or lateral incisors incomplete.

**Conclusion:**

Some overlapping and larger inclination in maxillary canine are features of normal eruption pattern at an earlier stage of canine development and while lateral incisor is incomplete in PTG (8.5–10.5 years). In addition to the overlapping and inclination, stages of canine and lateral incisor root as well as dental age should be observed radiologically when evaluating erupting maxillary canine in children of this age.

## Introduction

Maxillary canine eruption disturbances are common clinical problems in the developing permanent dentition. The movements of the erupting maxillary canines are unique and eruption disturbances can be partly explained by their long and complex eruption route. Overall, the prevalence of maxillary canine eruption disturbances is approximately 1.7% (Ericson and Kurol 1986).

The eruption of the permanent canines located in the maxillary bone begins when the crowns are ready and root development starts. On average, the permanent maxillary canines erupt into the oral cavity between the ages of 10–12 years (Haavikko 1970), but with a normal deviation of several years (Hurme 1949; Hägg and Taranger 1986). When a maxillary canine erupts normally into the oral cavity, the root is usually almost complete (Nolla 1960; Haavikko 1970). The manner of eruption of a maxillary canine is thought to be highly influenced by lateral incisor guidance (Broadbent 1941; Becker et al. 1981; Brin et al. 1986) as well as genetics (Peck et al. 1994; Baccetti 1998; Peck 2009) and the space available in the dental arch (Jacoby 1983).

Abnormal eruption of a maxillary canine can be verified from radiographic evidence after a clinical inspection has yielded negative or abnormal palpation findings. A panoramic radiograph (PTG) is the primary routine examination carried out in these cases. Initially, Ericson and Kurol (1988) defined a series of geometric measurements that could be made on PTGs to assist in diagnosing cases of a palatally displaced maxillary canine, after which various similar geometric measurements have been widely used to predict the eruption of maxillary canines (Lindauer et al. 1992; Power and Short 1993; Fernández et al. 1998; Warford et al. 2003; Chalakkal et al. 2011; Sajnani and King 2012; Alqerban et al. 2016; Naoumova and Kjellberg 2018). It has also been shown that these measurements can be used regardless of the labiopalatal position of the maxillary canine concerned (Sajnani and King 2012). The diagnosis of maxillary canine displacements from PTGs using geometric measurements is possible from the age of eight years onwards (Sajnani and King 2012).

According to earlier studies, overlapping of a permanent maxillary canine crown with the root of the adjacent lateral incisor can be considered normal up to 8 years of age (Fernández et al. 1998; Sajnani and King 2012) but should be considered a sign of displacement at the age of 9 years (Sajnani and King 2012) and especially by 10–12 years, when the maxillary canine should normally have erupted (Lindauer et al. 1992; Warford et al. 2003; Chalakkal et al. 2011). When the crown of a maxillary canine overlaps with the completed root of the adjacent lateral incisor in the PTG, this can be an early sign of an abnormal eruption (Fernández et al.^1998^).

During the normal maxillary canine eruption pattern, mesial inclination towards the maxillary midline increases until the age of eight to nine (Fernández et al. 1998; Sajnani and King 2012), after which the canine should begin to progressively upright itself. The inclination of an abnormally erupting maxillary canine has been shown to become more pronounced than that observed in normal eruption (Sajnani and King 2012).

The root development stage of a permanent tooth can be estimated from PTGs using for instance the method of Nolla (1960), but it has been shown that the root development stage does not differ between impacted and erupted maxillary canines (Sajnani and King 2012). Also, dental age should always be taken into account when evaluating the eruption of maxillary canines. This can be estimated using Demirjian’s method (Demirjian et al. 1973; Demirjian and Goldstein 1976), which scores the degree of development of seven teeth from the PTG. Method has been widely used in studies of pattern of growth (Chaillet et al. 2004; Baghdadi and Pani 2012). Dental age has been shown to be more advanced in girls at the mixed dentition (Demirjian and Levesque 1980; Chaillet et al. 2004).

The aim of the present work was to describe the variation in the eruption pattern of maxillary permanent canine as seen in a PTG in the late mixed stage of the dentition when the eruption to the oral cavity was later natural (no treatment). Our hypothesis was that some degree of overlapping of a canine crown with a lateral incisor root or mesial inclination of a canine is normal element to be found during the eruption of a maxillary canine.

## Materials and methods

This study is longitudinal and register-based. The material was gathered retrospectively at health centres in Eastern Finland and consisted of 1454 PTGs, primarily from third year primary school children born between 1980 and 1996. The PTGs had been taken in the radiological department of the health centre during annual oral check-ups for the purpose of examining the development of the permanent dentition in all cases. The two professionals who took most of the PTGs used Cranex DC 2 (Soredex) equipment until 15 March 2006 and a Planmeca Proline XC (Plandent) system after that.

The PTGs were copied digitally during the years 2006–2007 by RL and TK, who were also responsible for transferring the data to an Excel broadsheet. RL had been working as one of the health centre’s dentists at the time when the PTGs had been taken. Basic background information on the subjects was collected, including name, gender, date of birth and the date of the PTG. All available dental records and other information, including other PTGs and intraoral radiographs, were examined later during the years 2016–2020 by RL, JR, KK and WR.

Material based on PTGs has been used previously in several theses for a degree of Licentiate in Dentistry at University of Oulu, Finland. The following variables used in this report have been assessed as parts of these theses under the supervision of RL.

### Overlapping and inclination

The classifications of the overlapping and angle measurements derived from PTGs were assessed by TK using the neaView Radiology software (Neagen Oy). The measurements were performed on all four permanent canines (mandibular canines for later usage), and the exclusion criteria for this variable were emerged canine (based on PTG), complete primary dentition, complete permanent dentition, orthodontic treatment at the time of the PTG or poor quality of the PTG. Overlapping of maxillary canine was not determined from dentition with missing lateral incisor. The angle and overlapping measurements were assessed for repeatability by measuring 31 PTGs twice.

**Fig. 1:**
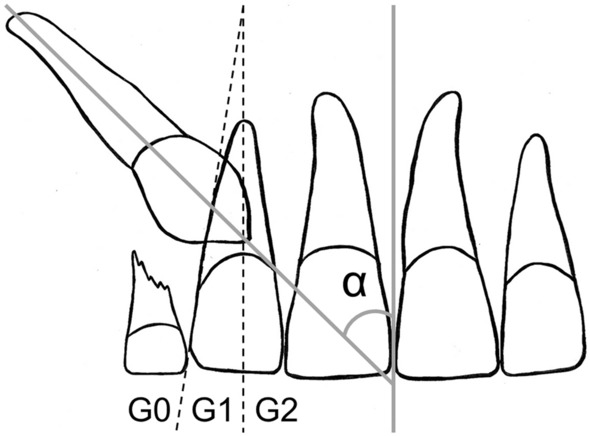
The maxillary permanent canine in relation to adjacent teeth. Measurements adapted from Ericson and Kurol (1988). The angle is that between the canine axis and the midsagittal suture of the maxilla (α). Grades of overlapping of the maxillary canine crown with the lateral incisor root: Grade 0, G0 (no overlapping); Grade 1, G1 (crown of the canine covering half or less of the width of the lateral incisor root); Grade 2, G2 (crown of the canine covering more than half of the width of the lateral incisor root)

Overlapping of a maxillary canine crown with the lateral incisor root (referred to simply as overlapping) was measured and classified Grade 0–2 (G0–G2). The inclination of the canine (inclination) was measured as the angle (*α*) between the midsagittal suture of the maxilla and the mid-axis of the canine, where the latter was defined by reference to the pulp chamber. In cases of crooked roots and rotated teeth, the average tooth axis had to be adjusted by reference to the crown and root (Fig. 1) (Ericson and Kurol 1988).

### Developmental stages of the maxillary canines and lateral incisors

**Fig. 2:**
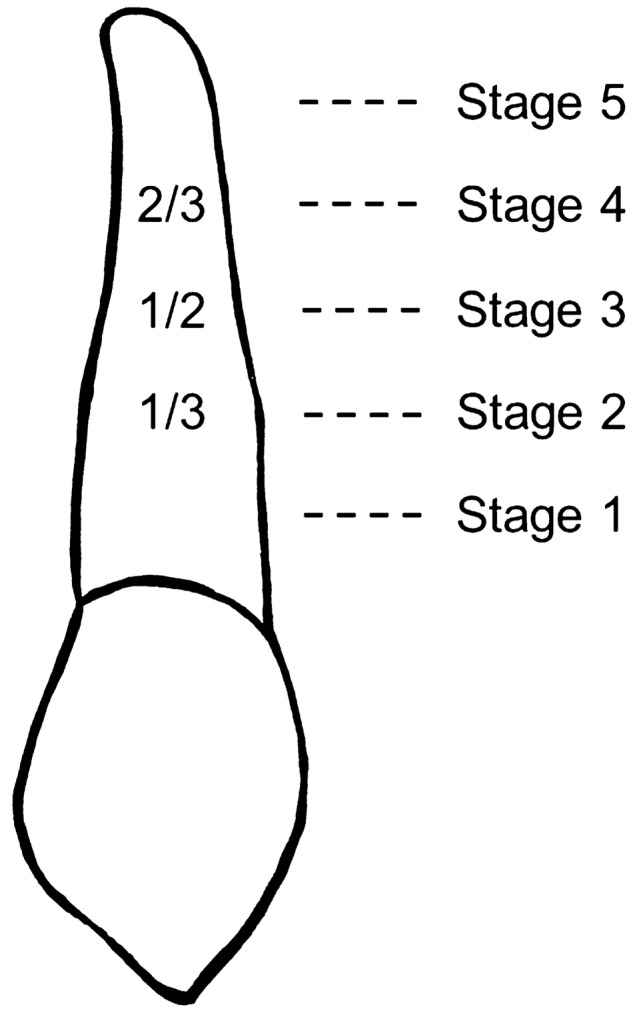
Stages of maxillary canine root development as first defined by Nolla (1960) and later grouped into five stages. Stage 1: root formation started (< 7.0); Stage 2: one-third of the root length completed (7.0, 7.2); Stage 3: half of the root length completed (7.5, 7.7); Stage 4: at least two-thirds of the root length completed (8.0–8.7); Stage 5: root completed, apex open/closed (9.0–10.0)

The root development stages of the maxillary canines were assessed from PTGs by WR using the method of Nolla (1960). The exclusion criteria for this variable were oligodontia (> six missing teeth) or an unreadable root due to overlapping structures visible in the PTG. Calibration was required before recording the final measurements. The development stage of the maxillary canine was measured by WR first from 655 PTGs and then from all 1454 PTGs (including the same 655 PTGs for a second time). Reliability was estimated by randomly selecting 65 PTG images (130 maxillary canines) from both data sets. The root development stages of the canines were divided into Stages 1–5 as shown in Fig. 2.

The development stages of the lateral incisors in the maxilla were assessed by WR from the PTG evidence simultaneously with the maxillary canines. The exclusion criteria were a peg-shaped lateral incisor, a missing lateral incisor, oligodontia (> six missing teeth) or an unreadable apex area of the lateral incisor due to overlapping structures or an artifact in the PTG. The development stage of each lateral incisor was categorized as incomplete (Nolla’s value 8.7 or less) or complete (Stage 5, Nolla’s value 9.0–10.0) by reference to Nolla (1960).

### Dental age

Dental age was analysed from PTGs by EM and JI using Demirjian’s dental maturity method (Demirjian et al. 1973; Demirjian and Goldstein 1976). The degrees of development of the individual teeth were evaluated and scored for seven teeth in the third quadrant from the PTG and then compared with Finnish maturity curves modified to conform to the same method (Chaillet et al. 2004). The exclusion criteria were a missing tooth (no counterpart) or poor quality of the PTG. Examiners 1 (EM) and 2 (JI) both assessed half of the material. To test the accuracy of the assessments, the two examiners assessed the same set of 30 PTGs two times, and their assessments were also compared with the results achieved by an orthodontist familiar with the method for calibration purpose. The child’s dental age was considered normal with mean ± 1SD (1SD = 1 year) and early or late outside the normal. For this report, dental age was grouped as either normal (early/normal) or delayed relative to the chronological age (Eskeli et al. 1999).

### Need for treatment

Need for treatment because of eruption of a maxillary canine was determined from all the dental records found in the health centre’s paper archives or software after PTG and until maxillary canine had been erupted by JR, RL and KK. The exclusion criteria were an emerged canine (based on PTG and/or dental records), poor quality of PTG, oligodontia (> six missing teeth), odontoma or a cyst in the maxillary canine area or transposition of a maxillary canine and the first premolar. “Natural eruption of a canine” was defined as no early or late treatment for maxillary canine during its eruption. Orthodontic treatment carried out before PTG or after maxillary canine had been erupted may have occurred.

This report is focused on the eruption pattern for a maxillary canine when no treatment was needed for either maxillary canine during eruption, a situation referred to here as ‘natural eruption of a canine’. The inclusion criteria for this study material were chronological age 8.5–10.5 at the time of the PTG, no missing teeth, no peg-shaped lateral incisors and no syndromes or clefts.

### Statistics

The results were recorded digitally using matrix software (Microsoft Excel) and the statistical analyses were performed using SPSS (version 26.0, IBM SPSS Statistics, Armonk, NY, USA). *P* values < 0.05 were considered statistically significant.

The repeatability of the assessments of overlapping and the root development stage of a maxillary canine was assessed using Cohen’s kappa. To check the reliability of the assessments of the inclination of a maxillary canine and the dental age, intra-class correlations (ICC) were calculated. The distributions of the variables examined were described in terms of frequencies and percentages, and comparisons of these variables between the genders were performed using Pearson’s Chi-square test. The same test was also used to analyse associations of stages in the root development of the maxillary canines, stages in the development of the lateral incisors and dental age with the grades of overlapping and with inclination in the natural eruption of the maxillary canines. Pearson’s Chi-square test was also used to compare distributions of the overlapping of a canine and the inclination of canines. The normality of continuous variables such as angles and chronological and dental ages was assessed visually using histograms and box plots. Gender-specific mean ages (chronological and dental) were analysed with the independent samples *t* test.

Comparisons of the mean angles between the root development stages of the maxillary canines within the overlapping groups were performed using one-way ANOVA and comparisons between the stages using Tukey’s post hoc test. The independent samples *t* test was used to compare the mean angles between the lateral incisor development groups and the dental age groups within the overlapping groups. The mean angles were also compared between the overlapping groups by applying the independent *t* test separately for the various stages of canine root development, the various lateral incisor development groups and the dental age groups.

### Ethics

This study was conducted retrospectively from data gathered from clinical dental records. Permission for copying the PTGs was given by the head of dental services in 2006 and permission for using the patient records and radiological images for the longitudinal setup was given first by the head of dental services in 2015 and later by the director of health services in 2019. The personal information gathered for this work was coded for the analyses by RL to prevent identification.

## Results

### Reliability

The intra-rater reliability for overlapping showed almost perfect agreement (d.13 kappa = 0.917, d.23 kappa = 0.849), and also for inclination (d.13 ICC = 0.933, d.23 ICC = 0.922), while that for the root development stage of the maxillary canine (Stages 1–5) showed substantial agreement (kappa = 0.777). Inter-rater reliability for dental age showed almost perfect agreement (ICC = 0.871), while intra-rater reliability showed substantial agreement for examiner 1 (ICC = 0.789) and almost perfect agreement for examiner 2 (ICC = 0.945). Thus, assessments performed here proved to be reliable in terms of their repeatability.

### Descriptive statistics

**Fig. 3:**
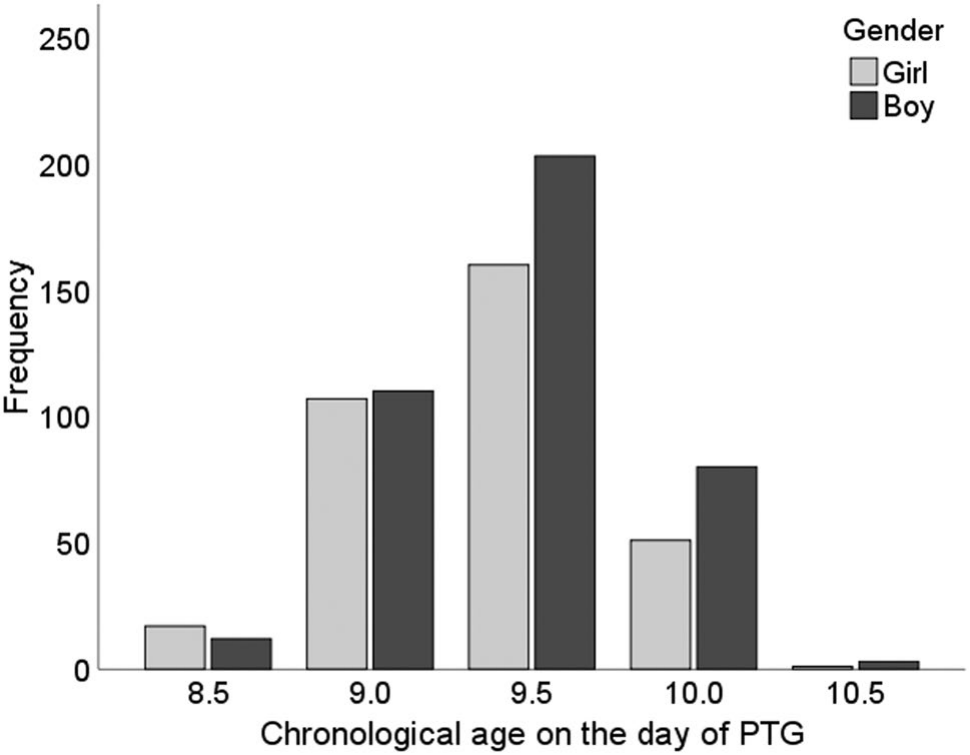
Distribution of children with natural eruption of a canine by chronological age

The material consisted of 1454 panoramic radiographs including 2907 maxillary canines (one congenitally missing right maxillary canine), the mean age of the children concerned at the time of PTG being 9.3 years (SD 0.6), a figure which did not differ between the genders (*p* = 0.067). Assessment of the need for treatment in the case of 1962 maxillary canines showed that almost all the maxillary canines (88.4%, *n* = 1734) erupted into the oral cavity without any treatment. The subsample representing natural eruption of a canine consisted of 744 panoramic radiographs (336 girls and 408 boys) including 1488 maxillary canines, and the mean age of these children at the time of PTG was 9.4 years (SD 0.4). The mean age differed significantly between the genders, being 9.4 years (SD 0.4) for the girls and 9.5 years (SD 0.4) for the boys, *p* = 0.001 (Fig. 3).

Altogether 1420 naturally erupting maxillary canines could be assessed for overlapping, and in 56.2% (*n* = 798) of these cases no overlapping (Grade 0) was detected in PTGs taken at the age of 8.5–10.5 years. Some overlapping (Grade 1) was detected in 41.8% of the canines (*n* = 594) and clear overlapping (Grade 2) in 2.0% (*n* = 28). The inclination of 1423 canines in the group with natural eruption could be measured, giving a mean angle of 12.4° (SD 7.4). The inclinations were under 25° in 94.5% (*n* = 1345) of the cases at the age of 8.5–10.5 years and ≥ 25° in 5.5% (*n* = 78) (Table 1).

**Table 1:** Distribution of variables in the group of natural eruption of maxillary canines and in the total material of PTGs

	Natural eruption of maxillary canines		Total study material	
	*n*	(%)	*n*	(%)
Overlapping of canine^a^				
Grade 0	798	(56.2)	1239	(54.5)
Grade 1	594	(41.8)	964	(42.4)
Grade 2	28	(2.0)	72	(3.2)
Total	1420	(100.0)	2275	(100.0)
Inclination of canine (°)				
< 15	938	(65.9)	1493	(65.0)
15–19.9	273	(19.2)	430	(18.7)
20–24.9	134	(9.4)	234	(10.2)
≥ 25	78	(5.5)	139	(6.1)
Total	1423	(100.0)	2296	(100.0)
Canine root development^b^				
Stage 1	93	(6.3)	314	(10.8)
Stage 2	856	(57.6)	1558	(53.8)
Stage 3	396	(26.6)	630	(21.8)
Stage 4	142	(9.5)	393	(13.6)
Total	1487	(100.0)	2895	(100.0)
Lateral incisor development^b^				
Incomplete	1146	(78.5)	2196	(77.8)
Complete	314	(21.5)	626	(22.2)
Total	1460	(100.0)	2822	(100.0)
Dental age^c^				
Normal	673	(95.2)	1202	(92.5)
Delayed	34	(4.8)	97	(7.5)
Total	707	(100.0)	1299	(100.0)

^a^Grade 0 (no overlapping), Grade 1 (≤ ½ overlapping) and Grade 2 (> ½ overlapping)

^b^Division is based on developmental stages of Nolla’s method (1960)

^c^Dental age is assessed for children by Demirjian’s method (Demirjian et al. 1973; Demirjian and Goldstein 1976)

**Fig. 4:**
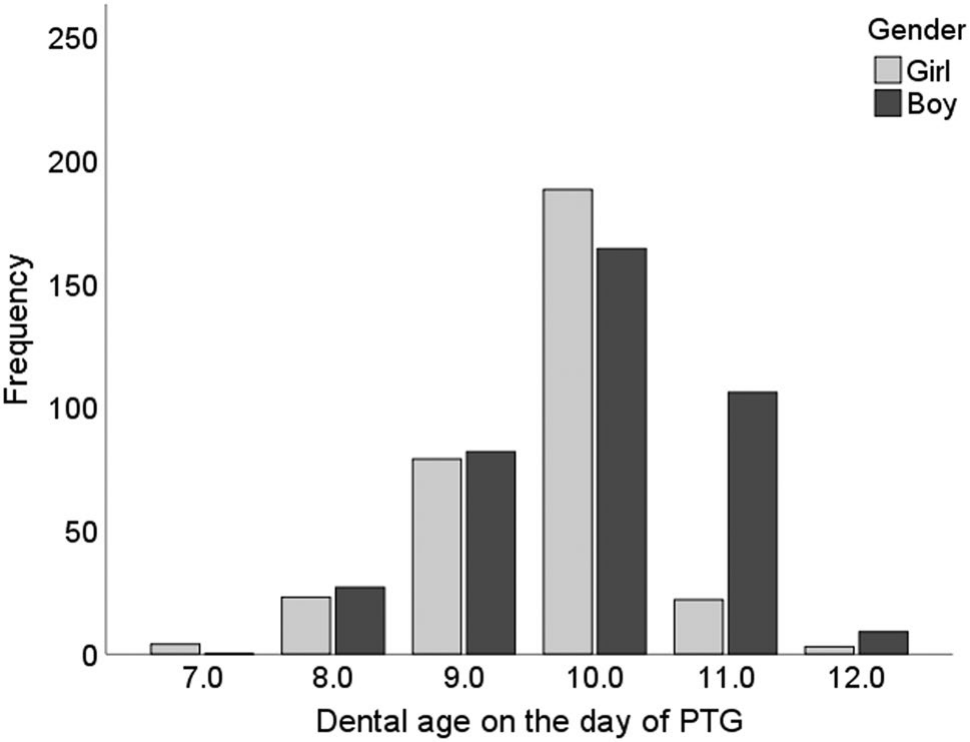
Distribution of children with natural eruption of a canine by dental age

The stage of root development could be assessed for 1487 naturally erupting maxillary canines, yielding values in the range 6.2–8.7 by Nolla’s method (1960). The majority (84.2%, *n* = 1252) were at Stages 2 and 3 at the age of 8.5–10.5 years (Fig. 2). Correspondingly, the stage of lateral incisor development, assessed from 1460 teeth, was incomplete in 78.5% of cases (*n* = 1146) and complete in 21.5% (*n* = 314) (Table 1). There was a statistically significant difference (*p* < 0.001) in the stage of canine and lateral incisor development between the genders, root development of the canines being more advanced the girls, who also had a complete lateral incisor more often than did the boys. No comparable statistical difference was found in overlapping (*p* = 0.12) or inclination (*p* = 0.554). Dental age could be assessed from 707 PTGs, giving a mean dental age of 9.8 years (SD 0.8). This again differed significantly between the genders, being 9.6 years (SD 0.8) for the girls and 10.0 years for the boys (SD 0.9), *p* < 0.001 (Fig. 4). Dental age was found to be normal in 95.2% cases (*n* = 673) and delayed in only 4.8% (*n* = 34), with no difference between the genders (*p* = 0.196) (Table 1).

### Comparisons between the variables

The grades of overlapping (Grades 0, 1, 2) in naturally erupting canines were compared to stages of canine and lateral incisor development (Table 2). Results pointed to statistically significant differences (*p* = 0.007) in the stages of canine root development according to the grade of overlapping. Overlapping occurred more often when root development in the canine was under halfway (Stages 1 and 2), and most often clear overlapping (4.3%, *n* = 4) was observed when the root was less than third of its final length (Stage 1). Statistically significant differences (*p* = 0.01) were also seen in the development stages of the lateral incisor according to the grade of overlapping, with at least some overlapping in 45.6% (*n* = 509) and 36.0% (*n* = 101) of the cases when the lateral incisor was incomplete and completed, respectively.

**Table 2:** Stages in root development of maxillary canine and lateral incisor and dental age vs. overlapping of canines with the lateral incisor root in the natural eruption of maxillary canines

	Grade 0^c^		Grade 1^c^		Grade 2^c^		Total	*P* value^d^
	*n*	(%)	*n*	(%)	*n*	(%)	*n*	
Canine root development^a^								
Stage 1	52	(55.9)	37	(39.8)	4	(4.3)	93	
Stage 2	436	(52.3)	379	(45.5)	18	(2.2)	833	
Stage 3	234	(62.1)	138	(36.6)	5	(1.3)	377	
Stage 4	76	(65.5)	39	(33.6)	1	(0.9)	116	
Total	798	(56.2)	593	(41.8)	28	(2.0)	1419	0.007
Lateral incisor development^a^								
Incomplete	607	(54.4)	484	(43.4)	25	(2.2)	1116	
Complete	180	(64.1)	98	(34.9)	3	(1.1)	281	
Total	787	(56.3)	582	(41.7)	28	(2.0)	1397	0.01
Dental age^b^								
Normal	720	(56.2)	536	(41.8)	25	(2.0)	1281	
Delayed	32	(47.1)	33	(48.5)	3	(4.4)	68	
Total	752	(55.7)	569	(42.2)	28	(2.1)	1349	0.171

^a^Division is based on developmental stages of Nolla’s method (1960)

^b^Dental age is assessed for children by Demirjian’s method (Demirjian et al. 1973; Demirjian and Goldstein 1976) and it was same for both maxillary canines

^c^Grade 0 (no overlapping), Grade 1 (≤ ½ overlapping) and Grade 2 (> ½ overlapping)

^d^Pearson’s Chi-square test

Comparison of the inclination of naturally erupting canines with the stages of development of the canine revealed statistically highly significant differences (*p* < 0.001) (Table 3), with the inclination of the maxillary canine being greater in the earlier stages of root development (Stages 1 and 2) and significantly (*p* < 0.001) greater when the root was third of its final length (Stage 2) if overlapping occurred (Table 4). At the other stages than Stage 2 in maxillary canine development there were no significant differences. As root development progressed from Stage 2 to halfway (Stage 3) the inclination decreased significantly (*p* < 0.001) regardless of the overlapping (Table 4), but there were no corresponding significant differences between the other root development stages.

**Table 3:** Stages in root development of maxillary canine and lateral incisor and dental age vs. inclination (°) in the natural eruption of maxillary canines

	< 15		15–19.9		20–24.9		≥ 25		Total	*P* value^c^
	*n*	(%)	*n*	(%)	*n*	(%)	*n*	(%)	*n*	
Canine root development^a^										
Stage 1	55	(59.1)	19	(20.4)	13	(14.0)	6	(6.5)	93	
Stage 2	492	(58.6)	189	(22.5)	96	(11.4)	63	(7.5)	840	
Stage 3	301	(80.1)	53	(14.1)	15	(4.0)	7	(1.9)	376	
Stage 4	90	(79.6)	12	(10.6)	9	(8.0)	2	(1.8)	113	
Total	938	(66.0)	273	(19.2)	133	(9.4)	78	(5.5)	1422	< 0.001
Lateral incisor development^a^										
Incomplete	692	(61.8)	240	(21.4)	118	(10.5)	69	(6.2)	1119	
Complete	232	(83.2)	26	(9.3)	15	(5.4)	6	(2.2)	279	
Total	924	(66.1)	266	(19.0)	133	(9.5)	75	(5.4)	1398	< 0.001
Dental age^b^										
Normal	860	(67.0)	241	(18.8)	116	(9.0)	67	(5.2)	1284	
Delayed	33	(48.5)	20	(29.4)	10	(14.7)	5	(7.4)	68	
Total	893	(66.1)	261	(19.3)	126	(9.3)	72	(5.3)	1352	0.019

^a^Division is based on developmental stages of Nolla’s method (Nolla 1960)

^b^Dental age is assessed for children by Demirjian’s method (Demirjian et al. 1973; Demirjian and Goldstein 1976) and it was same for both maxillary canines

^c^Pearson’s Chi-square test

**Table 4:** Mean inclination angle of maxillary canines (°) as a function of overlapping with a lateral incisor root, canine and lateral incisor development stages, and dental age in the natural eruption of maxillary canines

	Grade 0^c^			Grade 1 or 2^c^			*P* value^d^
	*n*	Mean angle	SD	*n*	Mean angle	SD	
Canine root development^a^							
Stage 1	52	13.8	8.0	41	13.3	7.7	0.776
Stage 2	432	13.0	7.4	396	14.8	6.9	< 0.001
Stage 3	231	9.5	6.8	143	10.4	6.4	0.210
Stage 4	75	8.0	7.1	38	10.6	8.0	0.081
*P* value^e^	< 0.001			< 0.001			
Lateral incisor development^a^							
Incomplete	600	12.4	7.4	508	14.3	6.9	< 0.001
Complete	179	8.8	7.4	99	9.3	6.6	0.597
*P* value^d^	< 0.001			< 0.001			
Dental age^b^							
Normal	712	11.3	7.5	558	13.3	7.0	< 0.001
Delayed	32	15.4	6.1	36	15.9	7.9	0.788
*P* value^d^	0.002			0.034			

^a^Division is based on developmental stages of Nolla’s method (Nolla 1960)

^b^Dental age is assessed for children by Demirjian’s method (Demirjian et al. 1973; Demirjian and Goldstein 1976) and it was same for both maxillary canines

^c^Grade 0 (no overlapping), Grade 1 (≤ ½ overlapping) and Grade 2 (> ½ overlapping)

^d^Independent-samples t test

^e^One-way ANOVA

Inclination of the naturally erupting maxillary canines showed statistically significant differences between the grades of overlapping (Grade 0, 1, 2) (*p* = 0.033), with ≥ 15° inclinations occurring more frequently at clear overlapping (Table 5). Also, regardless of overlapping, the mean angle between the canine and the maxillary midline was significantly larger (*p* < 0.001) in the earlier stages of root development and/or when the lateral incisor was incomplete (Table 4).

**Table 5:** Overlapping of canines with the lateral incisor root vs. inclination (°) of canines towards the maxillary midline in the natural eruption of maxillary canines

Overlapping of canine^a^	< 15		15–19.9		20–24.9		≥ 25		Total	*P* value^b^
	*n*	(%)	*n*	(%)	*n*	(%)	*n*	(%)	*n*	
Grade 0	547	(69.2)	136	(17.2)	73	(9.2)	34	(4.3)	790	
Grade 1	370	(62.6)	129	(21.8)	55	(9.3)	37	(6.3)	591	
Grade 2	13	(46.4)	8	(28.6)	4	(14.3)	3	(10.7)	28	
Total	930	(66.0)	273	(19.4)	132	(9.4)	74	(5.3)	1409	0.033

^a^Grade 0 (no overlapping), Grade 1 (≤ ½ overlapping) and Grade 2 (> ½ overlapping)

^b^Pearson’s Chi-square test

The differences in the stages of lateral incisor development with inclination of a naturally erupting maxillary canine were statistically highly significant (*p* < 0.001), with larger inclinations during eruption occurring more often when development of the lateral incisor was incomplete (Table 3), and especially when overlapping occurred (Table 4). The mean inclination was smaller if the lateral incisor was complete, an effect that was not dependent on overlapping (*p* = 0.597).

Dental age was also compared between the grades of overlapping (Grade 0, 1, 2), but the differences between the groups were not statistically significant (*p* = 0.171) (Table 2), even though the differences in dental age with inclination were statistically significant (*p* = 0.019). Children with delayed dental age had a larger inclination in their developing maxillary canines and more frequently inclinations between 15° ≤ and < 25°. (Table 3). When the mean inclination was assessed according to normal dental age, it was significantly (*p* < 0.001) larger if overlapping of the maxillary canine with lateral incisor occurred, whereas there was no similar significance if dental age was considered to be delayed (*p* = 0.788). Regardless of overlapping, there were significantly larger inclinations of the maxillary canines (*p* = 0.002 and *p* = 0.034) if the dental age was considered to be delayed (Table 4).

## Discussion

Panoramic radiography is a primary routine examination for assessing the development of the dentition, and thus provides a general picture from which a dentist can evaluate the positions of the erupting maxillary canines. The aim of this work was to describe diagnostic indications by defining variation in the eruption pattern of maxillary permanent canines in the late mixed stage of the dentition based on a PTG taken a couple of years preceding their natural eruption into the oral cavity. The developmental positions of the maxillary canines were evaluated by means of geometric measurements made on the PTGs (Ericson and Kurol 1988) and compared with the stages of development of the canines and lateral incisors (Nolla 1960) and the dental age (Demirjian et al. 1973; Demirjian and Goldstein 1976).

Results show diagnostic indications for normal eruption of the maxillary canines based on a PTG in children aged 8.5–10.5 years. The permanent maxillary canines are known to erupt into the oral cavity between the ages of 10 and 12 years (Haavikko 1970). Thus, our results give a PTG view covering a couple of years prior to the time of normal eruption into the oral cavity. At an age of 9–10 years, the permanent maxillary canines should be clinically palpable (Koch et al. 2017), making this a critical age, since a dentist can predict the path of maxillary canine eruption by clinical palpation, resorting to a radiographic examination if necessary.

Our material is a representative sample of PTGs of the developing dentitions in Finnish children from a normal population, and a sample of considerable size compared with other studies of the same type (Fernández et al. 1998; Sajnani and King 2012). No detailed descriptive study of the natural eruption pattern of the maxillary canines has been published previously.

It is commonly seen in PTG that a developing maxillary canine is overlapping the root of the adjacent lateral incisor at some point during its long eruption path, and hence almost half of the naturally erupting maxillary canines studied here showed some degree of overlapping in the PTG at ages of 8.5–10.5 years. This result differs from earlier opinions that overlapping could be considered normal up to eight years of age (Fernández et al. 1998; Sajnani and King 2012) but constitutes a sign of displacement by the age of nine (Sajnani and King 2012).

The dental ages of the present children were estimated to be mainly early or normal, and in most cases the roots of the maxillary canines had developed to between one-third and a half of their final length by the ages of 8.5–10.5 years, whereas root development at this age was earlier reported to be mainly a half to three-quarters of the final length (Sajnani and King 2012). One-fifth of the lateral incisors in our material were assessed to be developmentally complete, which again differs from an earlier finding that more than half of the lateral incisors were completed in this age cohort (Fernández et al. 1998). The maturation norms quoted by Nolla (1960) show that development of the lateral incisor may still be continuing at ages of 8.5–10.5 years.

Formation of the maxillary canines and lateral incisors in terms of root development stages was considered here to be more advanced in girls, confirming the general opinion regarding gender differences (Hurme 1949; Nolla 1960; Haavikko 1970; Hägg and Taranger 1986; Eskeli et al. 1999). However, the dental age of the girls in this chronological age cohort (8.5–10.5 years) was considered later than that of the boys, in contrast to earlier studies (Demirjian and Levesque 1980; Chaillet et al. 2004), although the grouped dental ages (normal and delayed) did not differ between the genders. This may reflect the nature of root development in terms of stages, such as fluctuations between latent and growth periods, and the roles of these in root formation in individual teeth.

We found here that overlapping of a maxillary canine crown with the root of a lateral incisor was more likely when root development in the maxillary canine had proceeded less than halfway. Beyond that point overlapping decreased. Overlapping occurred significantly less frequently if the lateral incisor was assessed to be complete, however, in our PTG findings a naturally erupting maxillary canine overlapped with the root of the lateral incisor root in more than a third of the cases in which the lateral incisor was complete. It has been suggested earlier that overlapping of a maxillary canine with a fully developed lateral incisor in a PTG may be a sign of an abnormal canine eruption path (Fernández et al. 1998). It should be noted that the present population was limited in age to 8.5–10.5 years, when the canines should already be palpable in the labial sulcus, whereas the population studied previously was aged 4–12 years (Fernández et al. 1998). According to our results, some degree of overlapping with the root of the lateral incisor at the age of 8.5–10.5 years can be considered a feature of the normal eruption pattern if root development in the maxillary canine is at an earlier stage. When development of the lateral incisor is complete, overlapping can occur during normal eruption in some cases.

Inclination of a canine was larger at the early stages in root development and/or when the lateral incisor was incomplete regardless of overlapping, and inclination decreased significantly as root development continued from one-third to halfway despite overlapping. This can be considered one of the cut-off points in the eruption path of a maxillary canine and is in line with earlier reports that canines upright themselves in time (Fernández et al. 1998; Sajnani and King 2012). Overlapping seems to increase the inclination significantly only when root development in the maxillary canine is one-third and/or the lateral incisor is incomplete. These findings suggest that larger inclination angles in a maxillary canine occur more frequently at the earlier stages of root formation and seem to be features of the normal pattern of maxillary canine eruption.

When dental age was considered in relation to inclination and overlapping, inclination gave significant differences whereas overlapping did not. According to this result, more clinical attention should be paid to PTG evidence of inclination if dental age is also considered. Larger inclination can be considered normal if the dental age is delayed regardless of overlapping. Overlapping seems to increase the inclination significantly only if dental age is considered early or normal.

The present study design is retrospective, and the study material has been collected from dental records which can be considered as a limitation of this study. For longitudinal view, all the dental records were not found later in the health centre’s paper archives or software after the PTG. That may have affected the results. One limitation of present study was that some children had received orthodontic treatment before PTG, for the primary dentition or during the early mixed stage of the dentition, such as Quad Helix for posterior cross-bite, braces on the upper incisors to close a large median diastema, slicing of the primary canines mesially in the case of minimum space deficiency during the eruption of the lateral incisors, or elastics for first molar cross-bite.

Our hypothesis was that some degree of overlapping of a canine crown with a lateral incisor root or mesial inclination of a canine is normal element to be found during the eruption of a maxillary canine. Hereby, we conclude that our hypothesis can be accepted. In addition, these results underpin the importance to determine inclination in PTG more than earlier has been suggested as well as dental age need to be taken into account related to the clinical follow-up of the maxillary canine eruption.

The features of normal and abnormal patterns of maxillary canine eruption warrant further research. We intend in the future to look at the group of children based on PTGs at the age of 8.5–10.5 years who later were treated for the eruption of a maxillary canine, to be able to consider follow-up and start treatment early enough.

## Conclusions

- Maxillary canines tend to erupt within the dental arch, even when the eruption path deviates in PTG.
- In earlier stages of maxillary canine root development and while lateral incisor is incomplete, some degree of overlapping and marked inclination of a maxillary canine can be regarded as part of the normal eruption pattern at the age of 8.5–10.5 years as seen in PTG.
- Despite the overlapping in PTG, inclination angle of maxillary canine tends to be larger when dental age is considered delayed.
- When evaluating the positions of the erupting maxillary canines radiologically, development stages of permanent maxillary canine and lateral incisor as well as dental age should always be observed in children of this chronological age.

## Data Availability

Not applicable.
